# Inflammatory Burden Index as a promising new marker for predicting surgical and oncological outcomes in colorectal cancer

**DOI:** 10.1002/ags3.12829

**Published:** 2024-05-28

**Authors:** Shinji Yamashita, Yoshinaga Okugawa, Naru Mizuno, Hiroki Imaoka, Tadanobu Shimura, Takahito Kitajima, Mikio Kawamura, Yoshiki Okita, Masaki Ohi, Yuji Toiyama

**Affiliations:** ^1^ Division of Reparative Medicine, Department of Gastrointestinal and Pediatric Surgery, Institute of Life Sciences Mie University Graduate School of Medicine Tsu Japan; ^2^ Department of Genomic Medicine Mie University Hospital Tsu Japan

**Keywords:** colorectal cancer, inflammatory burden index, prognostic biomarker, propensity score matching analysis, surgical site infection

## Abstract

**Aims:**

The prognosis of colorectal cancer (CRC) has been historically reliant on the Tumor Node Metastasis (TNM) staging system, but there is variability in outcomes among patients at similar stages. Therefore, there is an urgent need for more robust biomarkers. The aim of this study was to assess the clinical feasibility of the recently reported Inflammatory Burden Index (IBI) for predicting short‐ and long‐term outcomes in patients with CRC.

**Methods:**

This was a retrospective observational study of 555 CRC patients undergoing surgery for primary tumor resection. We determined the prognostic value of preoperative IBI for disease‐free and overall survival, and its predictive value for perioperative risk of infectious complications, including surgical site infection.

**Results:**

Increased preoperative IBI was significantly associated with advanced disease stage and poor oncological outcome in CRC patients. Higher IBI was independently linked to poorer disease‐free and overall survival. Similar outcomes were observed in a subanalysis focused on high‐risk stage II and stage III CRC patients. Elevated preoperative IBI was significantly correlated with an increased risk of surgical site infection and other postoperative infectious complications. Propensity score‐matching analysis validated the impact of IBI on the prognosis in CRC patients.

**Conclusion:**

We established preoperative IBI as a valuable predictive biomarker for perioperative risks and oncological outcomes in CRC patients. Preoperative IBI is useful for designing effective perioperative management and postoperative oncological follow‐up.

## Graphical abstract

This study emphasized the clinical importance of preoperative the Inflammatory Burden Index (IBI) as a robust biomarker for predicting both short‐ and long‐term outcomes in patients with colorectal cancer (CRC). We demonstrated significant associations between elevated preoperative IBI, disease progression, and postoperative infectious complications, which suggests a vital role for IBI in enhancing patient stratification and guiding more tailored perioperative management of CRC.

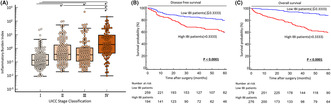

## 1 INTRODUCTION

Colorectal cancer (CRC) is one of the most commonly diagnosed malignancies worldwide, accounting for ~1 in 10 cancer cases and deaths.^1^ Despite improved diagnostic techniques and therapeutic regimens for CRC, >50% of patients are diagnosed at advanced stages.^2^ Recently, it has become clear that the incidence and mortality rates among younger individuals are on the rise, indicating a troubling trend that requires further investigation and attention.^1^, ^2^, ^3^ Historically, the prognosis of CRC has been primarily by the Tumor Node Metastasis (TNM) classification system established by the Union for International Cancer Control and the American Joint Committee on Cancer. TNM staging has been instrumental in guiding therapeutic decisions, but there can be significant variations in prognosis, even among patients at the same stage of the disease.^4^ Therefore, to improve the overall survival (OS) of CRC patients, robust biomarkers for predicting disease recurrence are necessary. Such biomarkers could facilitate the identification of high‐risk patients, enable meticulous postoperative monitoring, and aid in determining appropriate treatments.

Systemic inflammation, arising from host–tumor interactions, is currently recognized as the seventh hallmark of cancer and contributes to cancer incidence, stage, and progression.^5^, ^6^, ^7^ A growing body of research has highlighted the prognostic potential of systemic inflammatory markers in several types of cancer, including CRC. Systemic inflammation can be assessed using biochemical or hematological markers, such as C‐reactive protein (CRP),^8^ neutrophils,^9^ lymphocytes,^10^ albumin,^11^ and platelets,^12^ which are commonly measured in routine blood tests. Recently, numerous useful prognostic biomarkers in different types of cancer, including CRC, have been reported by combining these individual markers, such as the lymphocyte‐to‐CRP ratio,^7^ CRP‐to‐albumin ratio,^13^ neutrophil‐to‐lymphocyte ratio,^14^ and platelet‐to‐lymphocyte ratio.^15^ In addition, it has been frequently reported that postoperative complications affect long‐term oncological outcomes, with infectious complications, particularly surgical site infection (SSI), being the most significant.^16^, ^17^ Therefore, we posited that biomarkers capable of more accurately predicting oncological outcomes as well as postoperative infectious complications would be more sophisticated. In this study we focused on the recently reported Inflammatory Burden Index (IBI),^18^, ^19^ and assessed its practicality as a prognostic biomarker by evaluating its predictive capability for disease‐free survival (DFS) and OS. Furthermore, to confirm whether preoperative IBI has the potential to predict perioperative risks in CRC patients, we investigated the association between preoperative IBI and postoperative infectious complications.

## 2 METHODS

### 2.1 Patients and methods

This was a retrospective observational study. We enrolled 555 patients with CRC who underwent surgical treatment at our institution between January 2006 and December 2015. The TNM system was used for pathological staging of CRC.^20^ Resection of the primary tumor was performed in all patients and they were followed up for tumor recurrence at regular intervals for up to 5 y. No perioperative mortality was observed. During each annual hospital visit, all patients underwent chest X‐ray, colonoscopy, and abdominal computed tomography (CT).

The surgical approaches included laparotomy and laparoscopy with standard curative resection according to preoperative TNM staging. The diagnosis of CRC for all patients was based on pathological findings. After surgery, we recommended adjuvant chemotherapy for patients with stage IV, as well as for those with stage III and stage II with high‐risk factors.^21^ Among the patients with stage III and high‐risk stage II, 136 (50.7%) patients actually received chemotherapy. Patients were observed at 3‐mo intervals for 2 y after surgery, 6‐monthly for the subsequent 3 y, and annually thereafter. A medical history was taken and physical examination was conducted at each visit, and annual chest X‐ray, colonoscopy, and CT were performed. Data collected from inpatient and outpatient records included: demographic data (age and sex); preoperative factors (neoadjuvant therapy, preoperative complication); tumor‐specific details (histology, location, T classification, venous and lymphatic duct invasion, lymph node metastasis, distant metastasis); survival data (DFS and OS); and surgical details (surgical approach, operation time, and blood loss). In this study, there were 327 male and 228 female patients. The median age of the patients was 68 y (range, 27–94 y). Sixty‐eight (12.3%) patients had received chemotherapy and/or radiation therapy before surgery. Additionally, there were 41 (7.4%) patients with preoperative complications that could significantly impact systemic inflammation, such as bowel obstruction―defined as a situation requiring urgent decompression,^22^ perforation, and abscess formation. In many cases of bowel obstruction, emergency decompression was initially performed and was successful in several cases; therefore, only 13 (2.3%) patients across the entire group required emergency surgery. One hundred and fifty‐seven (28.3%) patients had stage I CRC, 149 (26.8%) had stage II, 147 (26.5%) had stage III, and 102 (18.4%) had stage IV. The median follow‐up time was 35.4 ± 28.1 mo. During the study period, 108 patients died from cancer‐related causes. Postoperative infectious complications as a short‐term outcome occurred within 30 d of surgery. Details of the infectious complications were obtained from medical records. Postoperative infectious complications included: wound infection (superficial or deep infection requiring treatment with antibiotics or wound drainage); intraabdominal abscess (intraabdominal fluid collection associated with fever or leukocytosis that discharged spontaneously or required surgical or radiologically guided drainage, with bacteria detected in blood or fluids culture); respiratory tract infection (respiratory symptoms and signs, and infiltration on chest radiography associated with fever or leukocytosis requiring antibiotic treatment); urinary tract infection; cholecystitis; and *Clostridium difficile*‐associated enteritis. SSI comprised wound infection and intraabdominal abscess, while remote infection included postoperative infectious complications other than superficial and organ space SSIs.

### 2.2 Laboratory measurement of preoperative IBI in routine blood tests

We obtained blood samples from each patient within 1 wk prior to surgical resection of their primary tumor. CRP and neutrophil and total lymphocyte counts were analyzed in routine blood tests, and we calculated IBI according to the formula: CRP (mg/dL) × neutrophils (/μL)/lymphocytes (/μL).

### 2.3 Propensity score matching

To minimize the effects of selection bias, propensity score matching (PSM) was implemented. High or low IBI in CRC patients was designated as the objective factor. The covariates used in building the propensity score were eight in total: sex (male or female), obstruction/perforation/abscess (present or absent), histological type (differentiated or undifferentiated), T classification (T1/2 or T3/4), venous invasion (present or absent), lymphatic invasion (present or absent), lymph node metastasis (present or absent), and distant metastasis (present or absent). We performed a one‐to‐one matching analysis between the two groups based on the estimated propensity score of each patient using a logistic regression model. A caliper width of 0.2 of the standard deviation of the propensity score was used for the one‐to‐one matching analysis.

### 2.4 Statistical methods

Statistical analysis was performed using MedCalc v. 16.8.4 (Mariakerke, Belgium). Results are expressed as median and interquartile range. Differences between groups were estimated using the Mann–Whitney *U* test or Kruskal–Wallis test. Receiver operating characteristic (ROC) curves with Youden's Index correction were established to determine optimal IBI cutoff thresholds for each outcome including survival outcome and SSI. For time‐to‐event analyses, survival estimates were calculated using Kaplan–Meier analysis, and groups were compared with the log‐rank test. DFS was measured from the date the patient underwent curative surgery to the date of disease recurrence, death from any cause (ie, cancer‐unrelated deaths were not censored), or until the last contact with the patient. OS was measured from the date the patient underwent surgery until the date of death from any cause (ie, cancer‐unrelated deaths were not censored) or the last known follow‐up for patients who were still alive. Cox's proportional hazards models were used to estimate hazard ratios (HRs) for recurrence or death. Following univariate analysis, variables with *p* < 0.05 were selected for multivariate analysis using the Cox proportional hazards regression model. The assumption of proportionality was confirmed for the Cox proportional hazards analyses by generating Kaplan–Meier survival curves (eg, high IBI vs low IBI) and ensuring that the two curves did not intersect each other. Multivariate logistic regression models were used to predict factors influencing postoperative infectious complications. All *p* values were two‐sided, and *p* < 0.05 was considered statistically significant.

## 3 RESULTS

### 3.1 Increased preoperative IBI significantly associated with CRC development

**FIGURE 1:**
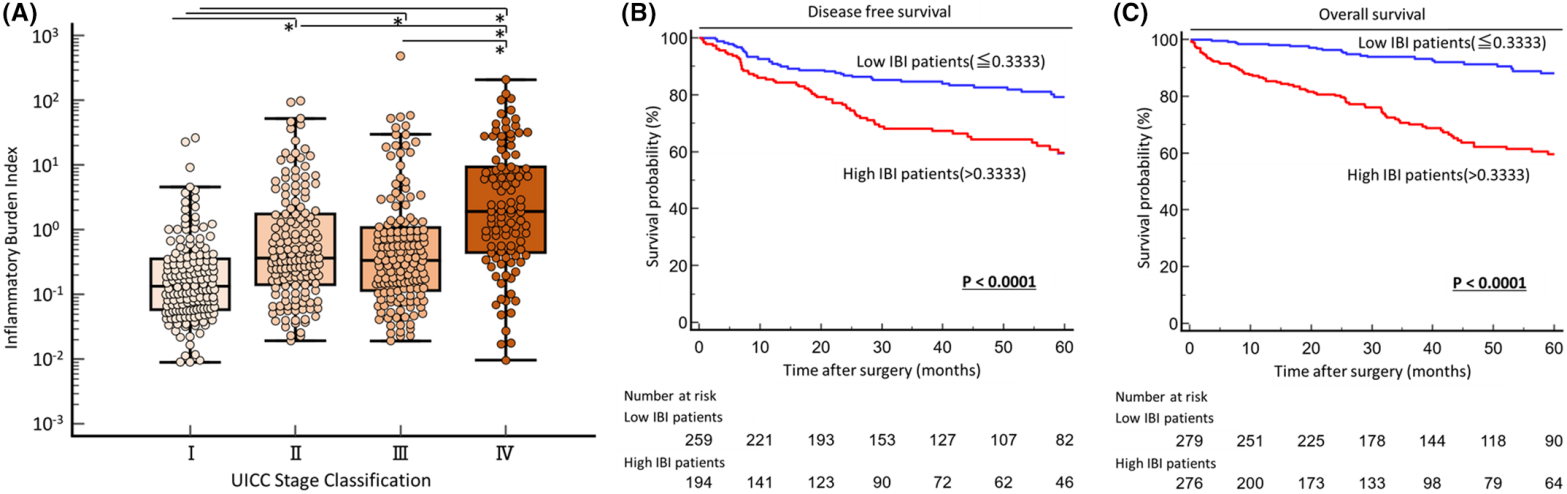
Clinical significance and prognostic impact of preoperative Inflammatory Burden Index (IBI) in colorectal cancer (CRC) patients. (A) Scattergrams of preoperative IBI according to TNM stage in CRC patients. Preoperative IBI was significantly increased in a stage‐dependent manner (*p < 0.05). Prognostic impact of IBI for disease‐free survival (DFS) (B) and overall survival (OS) (C) of CRC patients. DFS and OS rates in CRC patients with high IBI were significantly lower than in patients with low IBI (both *p* < 0.0001, log‐rank test). All statistical tests were two‐sided.

We evaluated the association between clinicopathological factors and preoperative IBI in CRC patients. In addition to sex (male), obstruction/perforation/abscess (present) and location (colon), increased preoperative IBI was significantly associated with several clinicopathological factors related to disease progression, including undifferentiated histology (*p* < 0.0001), advanced T stage (*p* < 0.0001), venous invasion (*p* = 0.002), lymphatic vessel invasion (*p* < 0.0001), lymph node metastasis (*p* < 0.0001), distant metastasis (*p* < 0.0001), and advanced TNM stage (*p* < 0.0001) (Table 1, Figure 1A).

**TABLE 1:** Clinicopathological variables and preoperative IBI in CRC patients.

Variables	*N*	Preoperative IBI	*p* value
		Median (IQR)	
Sex			
Male	327	0.37 (0.12–1.37)	0.03^a^,*
Female	228	0.26 (0.08–1.53)	
Median age (y)			
>68	276	0.36 (0.11–1.33)	0.49^a^
≤68	279	0.29 (0.1–1.53)	
Neoadjuvant therapy			
Yes	68	0.39 (0.12–2.21)	0.33^a^
No	487	0.33 (0.1–1.39)	
Obstruction/perforation/abscess			
Present	41	6.84 (0.57–22.0)	<0.0001^a^,*
Absent	514	0.29 (0.09–1.23)	
Histological type			
Differentiated	502	0.3 (0.1–1.23)	<0.0001^a^,*
Undifferentiated	53	1.33 (0.33–20.1)	
Location			
Colon	339	0.4 (0.1–2.16)	0.02^a^,*
Rectum	216	0.27 (0.09–1.04)	
Pathological T category			
pT1/T2	189	0.15 (0.06–0.39)	<0.0001^a^,*
pT3/T4	366	0.56 (0.17–3.72)	
Venous invasion			
Present	269	0.42 (0.12–2.98)	0.002^a^,*
Absent	286	0.25 (0.08–1.02)	
Lymphatic invasion			
Present	367	0.44 (0.12–2.75)	<0.0001^a^,*
Absent	188	0.22 (0.08–0.77)	
Lymph node metastasis			
Present	225	0.58 (0.16–3.77)	<0.0001^a^,*
Absent	330	0.25 (0.08–0.89)	
Distant metastasis			
Present	102	1.92 (0.44–9.37)	<0.0001^a^,*
Absent	453	0.25 (0.08–0.9)	
UICC stage classification			
Stage I	157	0.13 (0.06–0.36)	<0.0001^b^,*
Stage II	149	0.37 (0.14–1.76)	
Stage III	147	0.34 (0.12–1.08)	
Stage IV	102	1.92 (0.44–9.37)	

Abbreviations: CRC, colorectal cancer; IBI, Inflammatory Burden Index; IQR, interquartile range.

^a^ Mann–Whitney test.

^b^ Kruskal–Wallis test.

* *p* < 0.05.

### 3.2 High preoperative IBI significantly associated with poor oncological outcome in CRC

We performed time‐to‐event analysis to evaluate the potential of preoperative IBI as a prognostic biomarker; thus, we generated Kaplan–Meier survival curves subdivided by IBI based on optimal cutoff values for OS using ROC curve analysis with Youden index (IBI 0.3333). Patients with increased IBI using the same cutoff thresholds had significantly poorer prognosis in terms of DFS (log‐rank test, *p* < 0.0001, Figure 1B) and OS (log‐rank test, *p* < 0.0001) (Figure 1C). Based on Cox univariate proportional hazards analysis, implementation of neoadjuvant therapy, rectal tumor location, advanced T classification (T3/T4), venous invasion, lymphatic vessel invasion, lymph node metastasis, and high preoperative IBI status were significantly associated with poor DFS. Upon multivariate analysis, high IBI was identified as an independent prognostic factor for DFS (HR: 1.75, 95% confidence interval [CI]: 1.16–2.64, *p* = 0.007) (Table 2), in addition to rectal tumor, venous invasion, and lymph node metastasis. In contrast, implementation of neoadjuvant therapy, the presence of specific preoperative complications (obstruction/perforation/abscess), undifferentiated histology, advanced T classification (T3/T4), venous invasion, lymphatic vessel invasion, lymph node metastasis, distant metastasis, and high IBI were significantly associated with poor OS. Multivariate Cox regression analysis revealed that high preoperative IBI was also an independent prognostic factor for poor OS (HR: 2.83, 95% CI: 1.71–4.67, *p* = 0.0001) (Table 3) in CRC patients.

**TABLE 2:** Multivariate analysis for predictors of disease‐free survival in CRC patients.

Variables	Univariate			Multivariate		
	HR	95% CI	*p* value	HR	95% CI	*p* value
Sex (Male)	1.47	0.98–2.21	0.07			
Age (>68 y^a^)	1	0.68–1.47	1			
Neoadjuvant therapy (yes)	2.01	1.19–3.38	0.009*	1.36	0.76–2.44	0.29
Obstruction/perforation/abscess (present)	1.65	0.8–3.41	0.17			
Histological type (undifferentiated)	1.34	0.70–2.57	0.38			
Location (rectum)	2.22	1.51–3.28	0.0001*	2.25	1.46–3.48	0.0003*
T classification (pT3/4)	3.17	1.96–5.12	<0.0001*	1.7	0.97–2.97	0.06
Venous invasion (present)	3.37	2.24–5.05	<0.0001*	2.15	1.37–3.36	0.0008*
Lymphatic invasion (present)	2.89	1.79–4.67	<0.0001*	1.61	0.96–2.71	0.07
Lymph node metastasis (present)	2.73	1.85–4.02	<0.0001*	1.65	1.08–2.5	0.02*
High IBI status (>0.3333^b^)	2.22	1.5–3.28	0.0001*	1.75	1.16–2.64	0.007*

Abbreviations: CI, confidence interval; CRC, colorectal cancer; HR, hazard ratio; IBI, Inflammatory Burden Index.

^a^ The median age at surgery was 68 y in this cohort.

^b^ Cutoff thresholds for IBI were determined by receiver operating characteristic curve analysis with Youden's index for overall survival for CRC patients.

* *p* < 0.05.

**TABLE 3:** Multivariate analysis for predictors of overall survival in CRC patients.

Variables	Univariate			Multivariate		
	HR	95% CI	*p* value	HR	95% CI	*p* value
Sex (male)	1.31	0.88–1.95	0.18			
Age (>68 y^a^)	0.97	0.66–1.42	0.86			
Neoadjuvant therapy (yes)	1.88	1.17–3.03	0.009*	1.17	0.7–1.93	0.55
Obstruction/perforation/abscess (present)	2.76	1.62–4.69	0.0002*	1.52	0.87–2.64	0.14
Histological type (undifferentiated)	3.06	1.89–4.93	<0.0001*	2.63	1.6–4.34	0.0001*
Location (rectum)	1.4	0.96–2.05	0.08			
T classification (pT3/4)	4.59	2.57–8.2	<0.0001*	1.3	0.66–2.53	0.45
Venous invasion (present)	3.29	2.17–4.98	<0.0001*	1.63	1.01–2.63	0.05*
Lymphatic invasion (present)	3.84	2.19–6.74	<0.0001*	1.48	0.78–2.8	0.23
Lymph node metastasis (present)	3.01	2.02–4.47	<0.0001*	1.09	0.7–1.7	0.7
Distant metastasis (present)	7.81	5.33–11.4	<0.0001*	4.51	2.91–7.01	<0.0001*
High IBI status (>0.3333^b^)	5.19	3.25–8.3	<0.0001*	2.83	1.71–4.67	0.0001*

Abbreviations: CI, confidence interval; CRC, colorectal cancer; HR, hazard ratio; IBI, Inflammatory Burden Index.

^a^ The median age at surgery was 68 y in this cohort.

^b^ Cutoff thresholds for IBI were determined by receiver operating characteristic curve analysis with Youden's index for overall survival for CRC patients.

* *p* < 0.05.

### 3.3 Elevated preoperative IBI significantly associated with CRC development in high‐risk stage II and stage III

To evaluate the further potential of preoperative IBI as a prognostic biomarker, we conducted an analysis focusing on CRC patients classified as high‐risk stage II and stage III. In this study, stage II patients who met any of the following criteria extracted from the NCCN guidelines^21^ were classified as high‐risk stage II: fewer than 12 assessed lymph nodes, T4 tumors, obstruction or perforation at diagnosis, vascular or lymphatic invasion, poor histological differentiation, or positive resection margins. Consequently, we identified 121 high‐risk stage II patients and, together with 147 stage III patients, analyzed a total cohort of 268 patients. In this patient group, when evaluating the association between clinicopathological factors and preoperative IBI, an increase in preoperative IBI was significantly associated with the presence of specific preoperative complications (obstruction/perforation/abscess) (*p* < 0.0001), undifferentiated histology (*p* = 0.006), and advanced T stage (*p* = 0.0009). However, no significant association was found with lymph node metastasis, which is decisive in staging (Table S1).

### 3.4 High preoperative IBI significantly associated with poor oncological outcome in high‐risk stage II and stage III CRC

We generated Kaplan–Meier survival curves subdivided by IBI, using ROC curve analysis and the Youden index to establish the optimal cutoff value for OS in this group, which was determined to be IBI 0.4736. In this group as well, patients with increased IBI had significantly poorer prognosis in terms of DFS (log‐rank test, *p* = 0.04) (Figure S1A) and OS (log‐rank test, *p* = 0.005) (Figure S1B). Based on Cox univariate analysis, high preoperative IBI status was significantly associated with poor DFS. Multivariate analysis confirmed high IBI as an independent prognostic factor for DFS (HR: 1.73, 95% CI: 1.11–2.71, *p* = 0.02) (Table S2). The analysis also revealed that high preoperative IBI status was significantly associated with poor OS, with multivariate analysis confirmed high IBI as an independent prognostic factor for OS (HR: 2.76, 95% CI: 1.4–5.42, *p* = 0.003) (Table S3).

### 3.5 PSM analysis validated the impact of increased preoperative IBI on prognosis of CRC

PSM analysis is a widely recognized statistical method used to address selection bias and differing patient characteristics, thereby enhancing the validity of nonrandomized observational studies.^23^ To clarify the potential of preoperative IBI as a prognostic biomarker in CRC patients, we performed PSM analysis and categorized 302 patients (151 in each group) for further analysis. The differences in patient characteristics between the high and low IBI groups that were present before PSM analysis were not observed after PSM analysis (Tables S4 and S5). Kaplan–Meier survival curve analysis demonstrated that high preoperative IBI was significantly associated with poor prognosis in terms of DFS (*p* = 0.02) and OS (*p* < 0.0001) (Figure S2A,B) in the PSM cohort. These findings clearly indicate that preoperative IBI might be used as a prognostic biomarker in CRC patients.

### 3.6 Correlation of preoperative IBI and postoperative infectious complications in CRC

**FIGURE 2:**
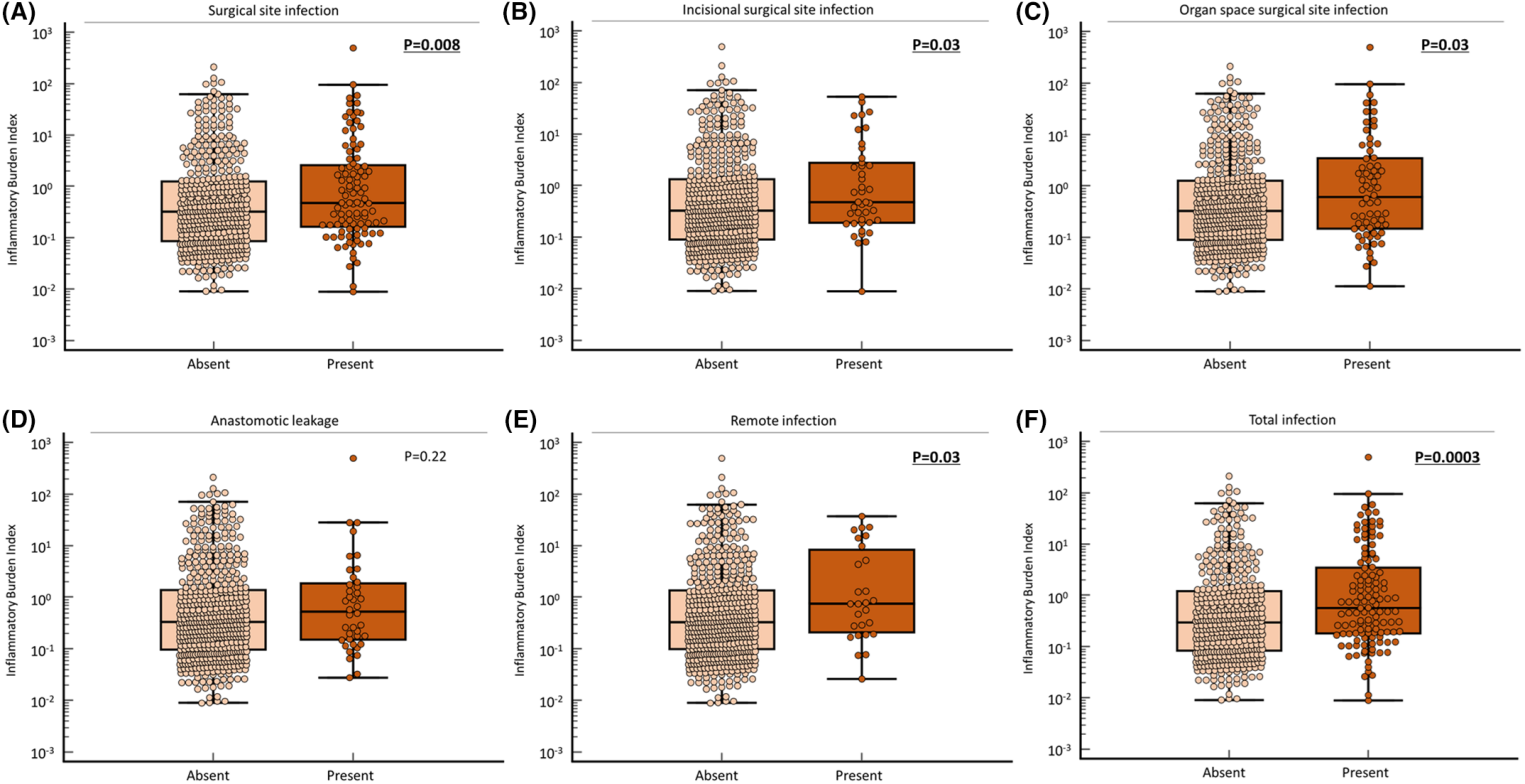
Preoperative Inflammatory Burden Index (IBI) was significantly higher in colorectal cancer (CRC) patients with postoperative infectious complications. Preoperative IBI was significantly higher in patients with surgical site infection (*p* = 0.008) (A) and its subcategories, incisional surgical site infection (B) and organ space surgical site infection (both *p* = 0.03) (C). Despite no significant correlation with anastomotic leakage (D), preoperative IBI was significantly elevated for remote infection (*p* = 0.03) (E), and a correlation was observed for total infection (*p* = 0.0003) (F), which included all complications related to infections. All statistical tests were two‐sided.

To determine the clinical impact of preoperative IBI as a predictive biomarker for postoperative complications in CRC patients, we evaluated the correlation between preoperative IBI and postoperative complications (Table 4, Figure 2). Preoperative IBI did not correlate with postoperative anastomotic leakage (*p* = 0.22); however, patients with SSI showed significantly higher preoperative IBI compared with those without such complications (*p* = 0.008). When SSI was subdivided and evaluated as incisional SSI and organ space SSI (both *p* = 0.03), a correlation with preoperative IBI was observed. Patients with remote infection had significantly elevated preoperative IBI (*p* = 0.03). In addition to SSI and remote infection, a correlation with preoperative IBI was confirmed for total infection, which included all infection‐related complications, such as systemic inflammatory response syndrome and bacteremia (*p* = 0.0003). To understand further the clinical impact of noninvasive biomarker potential for short‐term outcomes in CRC, we identified the optimal IBI cutoff value for SSI at 0.1025 using ROC curve analysis and Youden's index, and evaluated its predictive value with multivariate logistic analysis (Table 5). Long operation time (odds ratio [OR]: 1.83, 95% CI: 1.11–3.0) (*p* = 0.02) and increased IBI (OR: 1.91, 95% CI: 1.0–3.62) (*p* = 0.05) emerged as independent risk factors for SSI. Our data reveal that preoperative IBI could identify CRC patients who are at high risk for developing postoperative infectious complications.

**TABLE 4:** Infectious complications after operation and preoperative IBI in CRC patients.

Variables	*N*	Preoperative IBI	*p* value
		Median (IQR)	
SSI			
Present	100	0.48 (0.17–2.6)	0.008^a^ ^,^ *
Absent	455	0.32 (0.09–1.25)	
Incisional SSI			
Present	42	0.48 (0.19–2.76)	0.03^a^ ^,^ *
Absent	513	0.33 (0.09–1.32)	
Organ space SSI			
Present	68	0.62 (0.15–3.47)	0.03^a^ ^,^ *
Absent	487	0.33 (0.09–1.27)	
Anastomotic leakage			
Present	40	0.53 (0.15–1.86)	0.22^a^
Absent	515	0.33 (0.1–1.37)	
Remote infection			
Present	27	0.75 (0.21–8.61)	0.03^a^ ^,^ *
Absent	528	0.33 (0.1–1.36)	
Total infection			
Present	124	0.57 (0.18–3.47)	0.0003^a^ ^,^ *
Absent	431	0.3 (0.08–1.21)	

Abbreviations: CRC, colorectal cancer; IBI, Inflammatory Burden Index; IQR, interquartile range; SSI, surgical site infection.

^a^ Mann–Whitney test.

* *p* < 0.05.

**TABLE 5:** Multivariate analysis for predictors of surgical site infection in CRC patients.

Variables	Univariate			Multivariate		
	OR	95% CI	*p* value	OR	95% CI	*p* value
Sex (male)	0.95	0.62–1.48	0.84			
Age (>68 y^a^)	1.01	0.66–1.56	0.95			
Preoperative factor						
Neoadjuvant therapy (yes)	1.21	0.64–2.28	0.56			
Obstruction/perforation/abscess (present)	2.28	1.13–4.58	0.02*	1.57	0.75–3.28	0.23
Oncological factor						
Histological type (undifferentiated)	0.79	0.36–1.74	0.56			
Location (rectum)	1.06	0.68–1.64	0.81			
T classification (pT3/4)	1.49	0.92–2.42	0.1			
Venous invasion (present)	1.31	0.85–2.02	0.22			
Lymphatic invasion (present)	1.24	0.78–1.98	0.37			
Lymph node metastasis (present)	1.08	0.69–1.67	0.74			
Distant metastasis (present)	0.89	0.5–1.58	0.69			
Preoperative blood test						
High IBI status (>0.1025^b^)	2.56	1.38–4.75	0.003*	1.91	1.0–3.62	0.05*
Surgical factor						
Open versus laparoscopic surgery (open)	2.19	1.39–3.45	0.0008*	1.44	0.79–2.62	0.23
Operation time (>median)	2.13	1.35–3.34	0.001*	1.83	1.11–3.0	0.02*
Blood loss (>median)	2.67	1.68–4.23	<0.0001*	1.53	0.82–2.84	0.18

Abbreviations: CI, confidence interval; CRC, colorectal cancer; IBI, Inflammatory Burden Index; OR, odds ratio.

^a^ The median age at surgery was 68 y in this cohort.

^b^ Cutoff thresholds for IBI were determined by receiver operating characteristic curve analysis with Youden's index for the presence of SSl in CRC patients.

* *p* < 0.05.

## 4 DISCUSSION

The systemic inflammatory response associated with cancer is a pivotal sign of tumor progression. Many previous studies have identified serum systemic inflammatory indicators that have potential for prognostic predictions, such as interleukin‐1β^24^ and interleukin‐6,^25^ and these are considered to be closely involved in tumor proliferation and metastasis. In recent years, there has been an effort to create new indicators by combining existing biomarkers. We anticipate that understanding and combining the properties of each biomarker will lead to more accurate diagnoses, improved prognostic predictions, and better assessment of the risk of complications. Our team has previously reported on the utility of markers such as albumin‐to‐globulin ratio,^26^ lymphocyte‐to‐CRP ratio^7^ and modified Glasgow prognostic scale^27^ in CRC. These markers are readily available in routine blood tests for cancer patients.

IBI is a new indicator composed of serum CRP levels, neutrophil count, and lymphocyte count. Serum CRP,^28^ a prominent biomarker for systemic inflammation that is widely used in routine clinical practice, is associated with adverse outcomes in many types of cancer, including CRC. Neutrophils^29^ are abundant in peripheral blood and pivotal for defense against pathogens but they can also inadvertently support cancer progression when persistently activated. In cancer‐related inflammation, factors such as altered tumor cells and hypoxia perpetuate leukocyte recruitment, leading neutrophils to modulate the tissue microenvironment and inadvertently promote tumor development, angiogenesis, and metastasis. Lymphocytes^30^ have a critical role in the host cytotoxic immune response to tumors, and tumor‐infiltrating lymphocytes are widely recognized as key indicators of antitumor effects. Therefore, lymphopenia is recognized as an indicator of the host's immunological inadequacy in response to malignant disease and a prognostic marker for oncological outcome.^31^ Each variable reflects various aspects of the host's immunological response and systemic inflammation, and the combination of two variables, as well as each variable alone, may have the potential to predict prognosis.^14^, ^28^ However, individual markers can be influenced by a range of factors and may show variability that affects their reliability. Indeed, in this study CRP and neutrophil‐to‐lymphocyte ratio demonstrated high area under the curve (AUC) in ROC curve analysis for OS in CRC patients. However, the AUC value of preoperative IBI for survival was superior to those with a preoperative neutrophil‐to‐lymphocyte ratio in our cohort (data not shown). Furthermore, while CRP appeared to be comparable to IBI in AUC value for survival, a subanalysis limited to high‐risk stage II and stage III CRC patients indicated that it narrowly missed achieving statistical significance for DFS (data not shown). In contrast, preoperative IBI of incorporating neutrophils, lymphocytes, and CRP not only enables the depiction of the host's immunological response and systemic inflammation in a combined state, but also provides a more comprehensive, accurate, and reliable prognostic tool by integrating these three variables, thereby potentially offering superior predictive power for patient outcomes.

Several studies have verified the prognostic predictive ability of IBI in several types of cancer.^18^, ^19^ However, to the best of our knowledge, there has been a lack of studies focusing on prognostic prediction and perioperative complications of CRC patients. Thus, the present study explored whether preoperative IBI can be used as a predictive biomarker for the prognosis and perioperative risk in CRC patients. Our results demonstrated that high preoperative IBI was significantly associated with clinicopathological factors for disease development, and that CRC patients with high preoperative IBI showed poorer DFS and OS than those with low preoperative IBI. Furthermore, high preoperative IBI was an independent prognostic factor for DFS and OS. These findings are largely consistent with previous studies.^19^ Our study highlighted the following new insights. First, we conducted a subgroup analysis of high‐risk stage II and stage III CRC patients, demonstrating within this group that patients with high preoperative IBI showed poorer DFS and OS than those with low preoperative IBI. Furthermore, it was shown that high preoperative IBI is an independent prognostic factor for both DFS and OS in this group. Second, we performed PSM analysis to overcome the selection bias of patient characteristics and validated that CRC patients with high preoperative IBI had poorer DFS and OS than those with low preoperative IBI. Third, we have clearly demonstrated the relationship between preoperative IBI and postoperative infectious complications, and revealed that preoperative IBI was a potential predictor for postoperative SSI. From the above, patients with high preoperative IBI might benefit from more aggressive postoperative chemotherapy or more detailed follow‐up, beyond what is usually suggested by TNM classification strategies alone, thereby potentially improving prognosis. Additionally, since high preoperative IBI indicates an increased risk for SSI, these patients could potentially benefit from adjustments in perioperative management, such as the choice of antiseptic^32^ or method of closing the abdominal wall,^33^ and modifications to postoperative antibiotic therapy, including the choice and duration of medication. These changes might reduce the incidence of SSI. However, since the IBI cutoff value of 0.1025 for SSI risk is around the 25th percentile (1st IQR) of the patients who did not experience SSI, it is uncertain that this cutoff value works for the SSI predictor, even if statistically significant. On the other hand, the prolonged operative time, identified as another independent risk factor for SSI in this study, is a straightforward and empirically significant factor. In fact, simply screening patients based on the length of operation time as a single trigger for SSI measures might be sufficient to achieve significant effects. Although we anticipate that identifying patients with both high IBI and prolonged operative time will predict the occurrence of SSI more effectively, while narrowing down the number of patients, further prospective clinical trials are warranted to elucidate these findings, using these parameters with or without interventions such as specialized techniques for closing the abdominal wall to clarify these points.

Recently, the impact of postoperative complications on oncological prognosis has become increasingly evident. The Japan Society for Surgical Infection conducted a multicenter retrospective cohort study involving 1817 curative stage I/II/III CRC patients.^34^ They clearly demonstrated that postoperative infectious complications significantly correlated with a decrease in cancer‐specific survival and abolished the survival benefit of adjuvant chemotherapy. Similarly, a meta‐analysis of 154,981 patients identified a significant association between infectious complications, including SSI, and adverse oncological outcomes.^35^ The IBI we have recognized as valuable not only predicts prognosis but also suggests the potential to predict patients at high risk for infectious complications. Thus, from this perspective, the IBI could contribute to improvements in oncological outcomes beyond existing markers.

There were several limitations to the present study. First, this was a retrospective cohort study. Second, all of the patients were from a single institution in Japan. Third, the appropriateness of the IBI cutoff point remains uncertain. In fact, the IBI cutoff point identified in this study shows significant variation from those found in prior studies. This variation could be attributed to several factors, such as differences in patient selection, the heterogeneity of methods used to determine the cutoff values, and the variability in CRP units between countries and regions. To validate our findings, it is essential to conduct larger prospective trials that include patients from multiple centers, ensuring standardized patient selection and uniform application of statistical methods.

In conclusion, our study highlights the clinical utility of preoperative IBI as a predictive biomarker for postoperative infectious risk and oncological outcome in CRC patients. Assessment of preoperative IBI may help physicians to design more effective perioperative management and postoperative oncological follow‐up strategies for CRC patients.

## AUTHOR CONTRIBUTIONS

Study Design: Shinji Yamashita. Data collection: Shinji Yamashita, Yoshinaga Okugawa, Naru Mizuno, Hiroki Imaoka, Tadanobu Shimura, Takahito Kitajima, Mikio Kawamura, Yoshiki Okita, and Masaki Ohi. Statistical analysis and interpretation of results: Shinji Yamashita. Drafting of the article: Shinji Yamashita. Supervision: Yoshinaga Okugawa, and Yuji Toiyama.

## FUNDING INFORMATION

This study did not receive support from any organization.

## CONFLICT OF INTEREST STATEMENT

Yuji Toiyama is an editorial board member of *Annals of Gastroenterological Surgery*. The remaining authors declare no conflicts of interest for this article.

## ETHICS STATEMENT

Approval of the research protocol: This study was conducted in accordance with the ethical principles of the Declaration of Helsinki. This was a retrospective study approved by the Review Board of Mie University Hospital.

Informed Consent: Patients provided written informed consent.

Registry and the Registration No. of the study/trial: N/A.

Animal Studies: N/A.

## Supporting information


Figures S1–S2.

Tables S1–S5.
