# Supplementary figures and images for: Inflammatory Burden Index as a promising new marker for predicting surgical and oncological outcomes in colorectal cancer
Source: Ann Gastroenterol Surg. 2024 May 28;8(5):826–35. doi: 10.1002/ags3.12829 (PMC11368506; doi:10.1002/ags3.12829)

## Slide 1
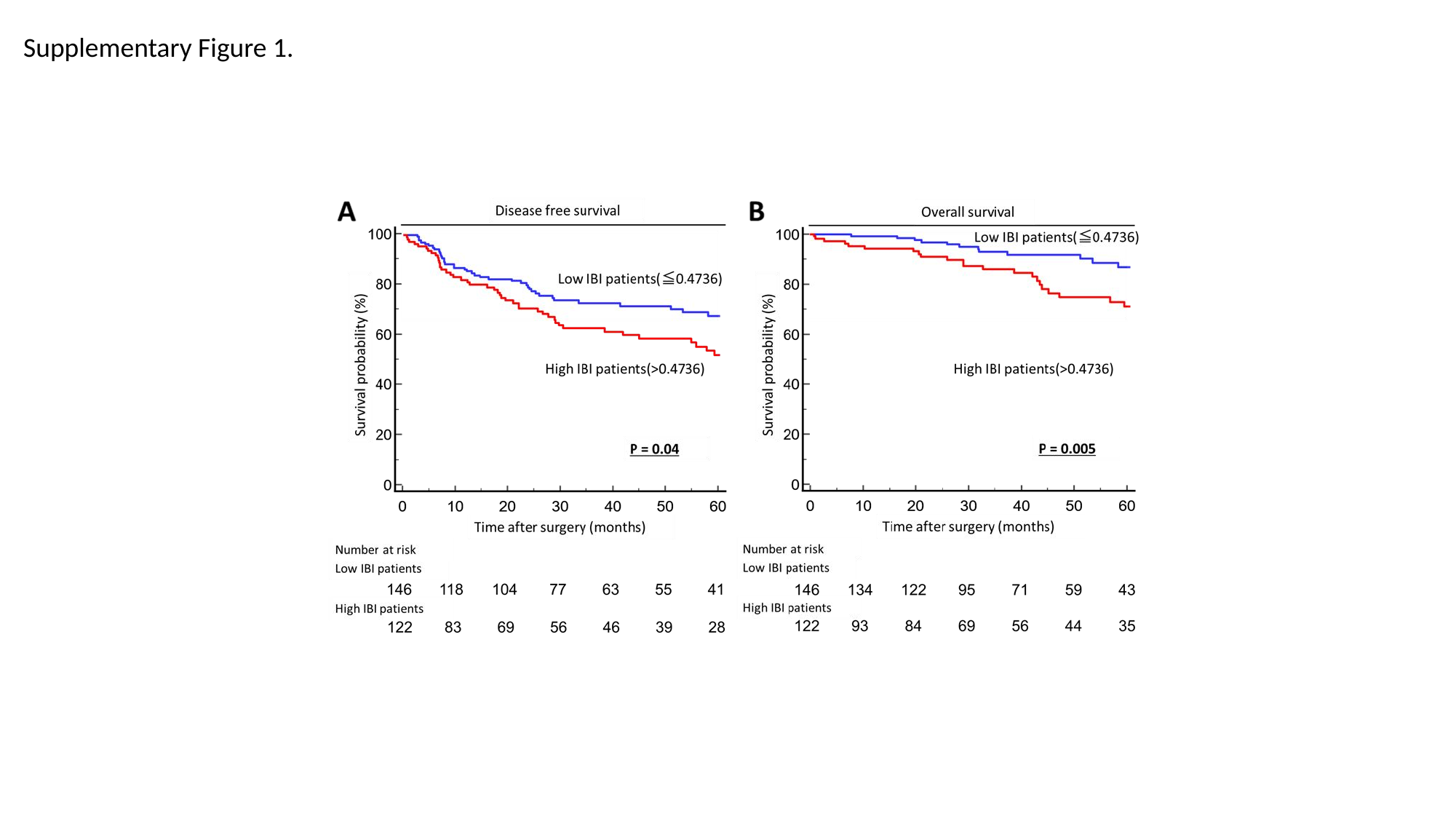

Supplementary Figure 1.

## Slide 2
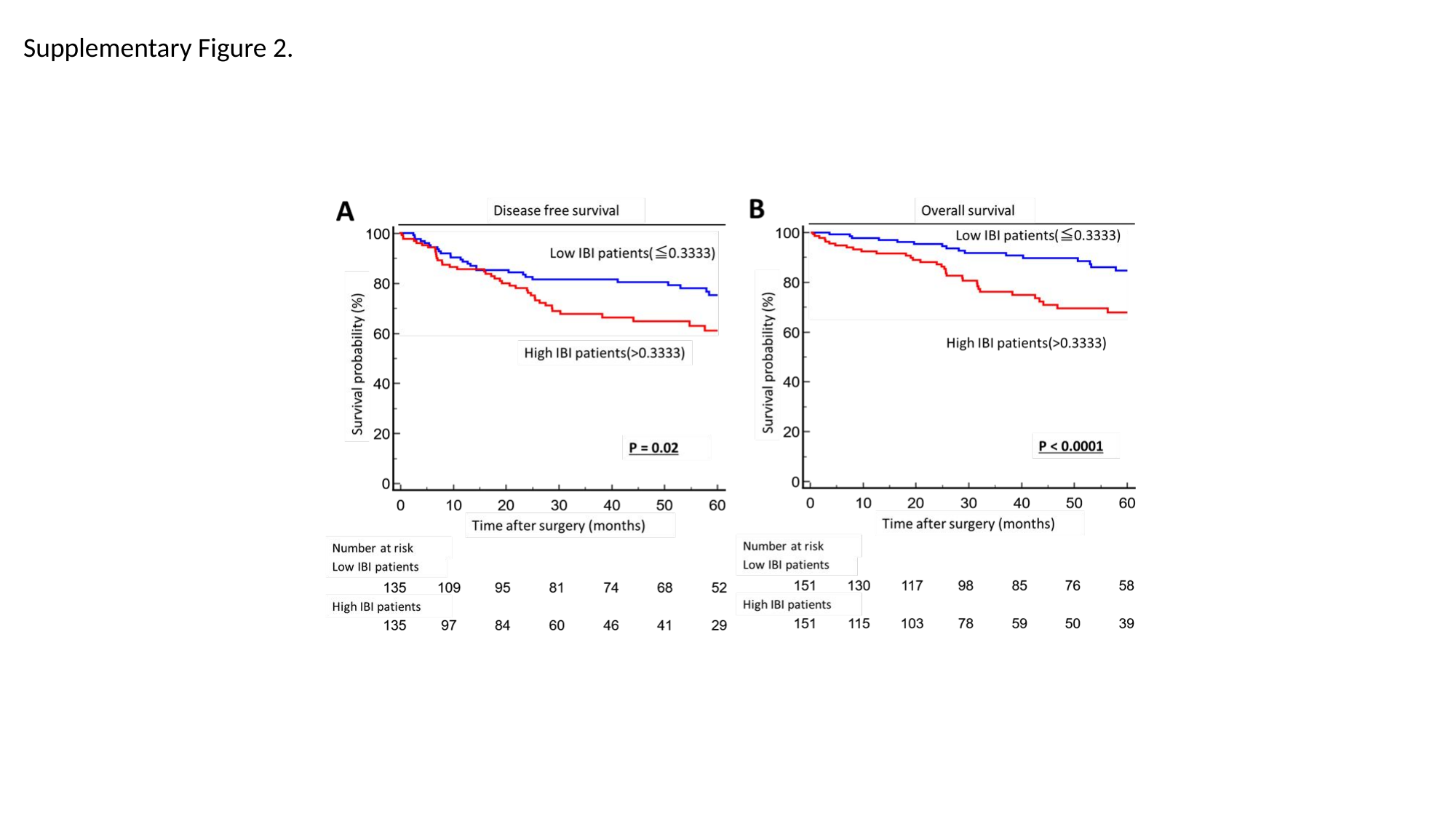

Supplementary Figure 2.

Supplement: Supplementary file 1 — Figures S1–S2. [file AGS3-8-826-s001.pptx]
